# The process of recovery in women who experienced psychosis following childbirth

**DOI:** 10.1186/1471-244X-13-341

**Published:** 2013-12-20

**Authors:** Laura McGrath, Sarah Peters, Angelika Wieck, Anja Wittkowski

**Affiliations:** 1 School of Psychological Sciences, the University of Manchester, 2nd Floor Zochonis Building, Brunswick Street, Manchester M13 9PL, UK; 2 Manchester Mental Health and Social Care Trust, Wythenshawe Hospital, Manchester, UK

**Keywords:** Puerperal psychosis, Postpartum psychosis, Postnatal psychosis, Recovery, Grounded theory, Qualitative, United Kingdom

## Abstract

**Background:**

Psychosis following childbirth affects 1–2 mothers per 1000 deliveries. Onset is rapid and functioning is severely affected. Although prognosis in terms of symptom remission is generally good, long-term disability can persist. The study’s aim was to develop a theoretical understanding of recovery from psychosis following childbirth.

**Methods:**

Semi-structured interviews were conducted with 12 women with experience of psychosis following childbirth. Interview transcripts were analysed using grounded theory methodology.

**Results:**

A theory of four superordinate themes was developed from the data, including: (i) the process of recovery; (ii) evolving an understanding; (iii) strategies for recovery; and (iv) sociocultural context. The process of recovery and women’s understanding of their experience were conceptualised as parallel processes, which informed one another. Women found that a diagnosis facilitated their use of particular strategies.

**Conclusions:**

This study highlighted a complex and ongoing process of recovery from psychosis following childbirth. Sensitivity to a woman’s position in the process of recovery has the potential to facilitate professionals in assessing readiness for different interventions which will be likely to result in women feeling more understood, accepted and supported.

## Background

Historically serious mental illnesses (SMI) were viewed as inevitably chronic and debilitating. Recovery was defined as an absence of symptoms and was considered unlikely. Services based on this model were seen as contributing to the chronicity of SMI via a number of social and interpersonal processes [1].

Published accounts of recovery by service users in the 1980s saw the birth of the recovery movement which has been gaining momentum ever since. Definitions came to include wider personal, psychological and social factors, which contribute to a fulfilling life even when symptoms or impairments remain [2]. The concept of recovery has been further promoted and has been influential in service and policy development [3]. However, difficulties have emerged in the development of recovery-focused services [4] and it has been suggested that the need for such services remains [5].

Psychosis following childbirth has been referred to in the literature as postpartum psychosis and puerperal psychosis (PP). It is a severe form of mental illness that affects 1–2 mothers per 1000 deliveries [6]. Onset is rapid and symptoms include hallucinations, delusions, mood swings, confused thinking and disorganised behaviour [7]. Although functioning is severely affected, with the potential to confer risk to the mother and infant, prognosis is good in terms of symptom remission [8].

There has been a lack of consensus regarding the classification of PP. Debate continues about whether psychosis following childbirth should be considered a distinct clinical entity with childbirth as the major aetiological agent [9] or whether childbirth is a trigger that exposes a pre-existing vulnerability to psychosis. At present neither the DSM IV-TR [10], nor the ICD-10 [11] recognise PP as a distinct entity. ICD-10 allows for a distinct puerperal diagnosis only if women cannot be diagnosed with another disorder. In light of the movement toward recovery-orientated services, there have been calls to operationalise the concept of recovery in terms of those who have experienced it [12], to investigate what facilitates recovery and develop outcomes, which are meaningful for service users.

### Aims of the study

Therefore, the aim of the current study was to develop a theoretical understanding of recovery from psychosis following childbirth. As women with PP experience ongoing difficulties, including anxiety and depressive symptoms, stigma and changes to their relationships, the researchers aimed at investigating their views using a recovery framework.

## Methods

This qualitative study employed constuctivist grounded theory methodology [13], because psychosis following childbirth is an under-theorised area and the study was discovery-orientated. The ‘constant comparison’ method was used to construct theory directly from the data.

Recruitment took place between April 2011 and May 2012. Ethical approval was obtained from the University of Manchester’s Research Governance Department, the local Research Ethics Committee (LREC reference: 11/H1003/8) and the relevant NHS Trust Research and Development Department.

### Participants

Women were recruited from a Mother and Baby Unit (MBU) in Northwest England and by advertisements placed on website forums and newsletters for women with experience of postnatal illness. Due to the contentious nosology associated with PP, women who experienced any form of psychosis following childbirth were included [14]. Inclusion criteria were i) over 18 years of age, ii) fluent in verbal and written English, and iii) a diagnosis of PP, any disorder in the schizophrenia category, a manic episode or bipolar affective disorder according to ICD-10. The inclusion criteria were broadly defined to gather diverse and heterogeneous viewpoints on the process of recovery. Therefore, women were sought from different locations with different experiences of local service provision at different stages of recovery. Where possible, diagnosis was verified by checking medical records but no formal psychiatric assessment were made. Information about symptoms and diagnosis was collected using a demographic questionnaire.

Potential participants were provided with a Participant Information Sheet detailing the aims and procedures involved. Once written informed consent was obtained, women were interviewed (by LM). The study maintained participants’ right to withdraw, confidentiality and anonymity regarding quotations.

Twelve participants agreed to be interviewed. Demographic characteristics are shown in Table 1. All women were White British, living in England or Wales. Women’s ages ranged from 26 to 45 years (mean 35.6) and they reported experiences of psychosis following childbirth which occurred between 4 months ago and 23 years, 4 months ago (mean 5 years, 6 months). Two women had subsequent pregnancies, neither of which resulted in a recurrence of psychosis. None of the women had experienced psychosis prior to this episode. Three women described a history of more common mental health problems (e.g., anxiety and depression). One participant had a previous psychiatric admission for severe depression prior to her psychotic episode. There was variability in the environments in which women were cared for following their episode of PP: four received care on a general psychiatric ward, two were initially admitted to a general psychiatric ward and then transferred to a Mother and Baby Unit (MBU) with their baby, two remained on maternity wards and one woman received care at home.

**Table 1:** Demographic information at time of interview

	**Maternal age range**	**Child’s age range in years**	**Employment status**	**Marital status**	**Diagnosis**	**Recruited from**
**1**	45-49	5-9	Full time	Single	Post partum depression with psychotic features	Advert
**2**	40-44	5-9	Part time	Married	Puerperal psychosis	Advert
**3**	25-29	1-4	Housewife and voluntary work	Cohabiting	Puerperal psychosis	Advert
**4**	30-34	Under a year	Part time	Married	Puerperal psychosis	Advert
**5**	50-54	20+	Part time	Married	Puerperal psychosis	Advert
**6**	30-34	1-4	Housewife	Married	Puerperal psychosis	Advert
**7**	30-34	1-4	Part time	Married	Puerperal psychosis	Advert
**8**	30-34	1-4	Housewife	Married	Puerperal psychosis	Advert
**9**	30-34	1-4	Part time	Married	Puerperal psychosis	MBU
**10**	40-44	15-19	Full time	Divorced	Puerperal psychosis	Advert
**11**	30-34	1-4	Full time	Married	Puerperal psychosis	Advert
**12**	30-34	Under a year	Maternity leave from full time employment	Married	Puerperal psychosis	MBU

### Interviews

Interviews were semi-structured using an interview schedule containing general open questions to guide areas of discussion relating to women’s experience of psychosis. Questioning was responsive to women’s comments and areas for discussion raised by women were explored further. Interviews were audio-recorded for the purpose of data analysis and duration of interviews ranged from 37 minutes to 110 minutes, with most lasting approximately an hour. Eleven women were interviewed face-to-face and one interview was conducted over the telephone due to geographical distance.

### Data analysis

Throughout the parallel processes of data collection and analysis, the following stages of data analysis led to refinements of the interview schedule on two occasions in order to develop the theory as it emerged: (i) data transcription, (ii) line-by-line coding, (iii) refinement of interview schedule, and (iv) focused coding.

### Credibility and trustworthiness

Qualitative guidelines [13,15] were used to ensure methodological rigour. In order to test credibility, the developing theory was refined by theoretical sampling, altering the interview schedule, making memos and having regular discussions within the research team. The interview schedule was refined on two occasions. Refinements included prompts to facilitate an exploration of the developing theory. The following prompt was included in the interview schedule prior to the fourth interview: “Were you discouraged from having another child?” Prior to the tenth interview, the schedule was again revised by including this prompt: “Any strategies used to stay well?”

Discussion with the research team enabled reflection upon potential sources of bias. In later interviews the developing theory was discussed with participants to find out whether it resonated with them. Transparency was ensured by the use of a reflective journal, memos and supervision. In the presentation of findings, direct quotes demonstrate that the analysis was fully grounded in participants’ accounts.

### Reflexivity

As the importance of reflexivity is emphasised in constructivist grounded theory [13], the main researcher (LM) considered her motives, background and role as a researcher and the ways in which experiences and knowledge might influence the generation, analysis and interpretation of data. She was a 28-year-old White British woman who had some experience of working with people with psychosis in the context of an Early Intervention in Psychosis service. A recovery approach, valued by service users, was one of the guiding principles used within such teams. Although she had no experience of working with someone who had experienced psychosis in the context of childbirth, she reflected upon the importance of considering the context in which psychosis was experienced and the effects not only for the person themselves but also their family at a time, expected to be joyful.

## Results

**Figure 1:**
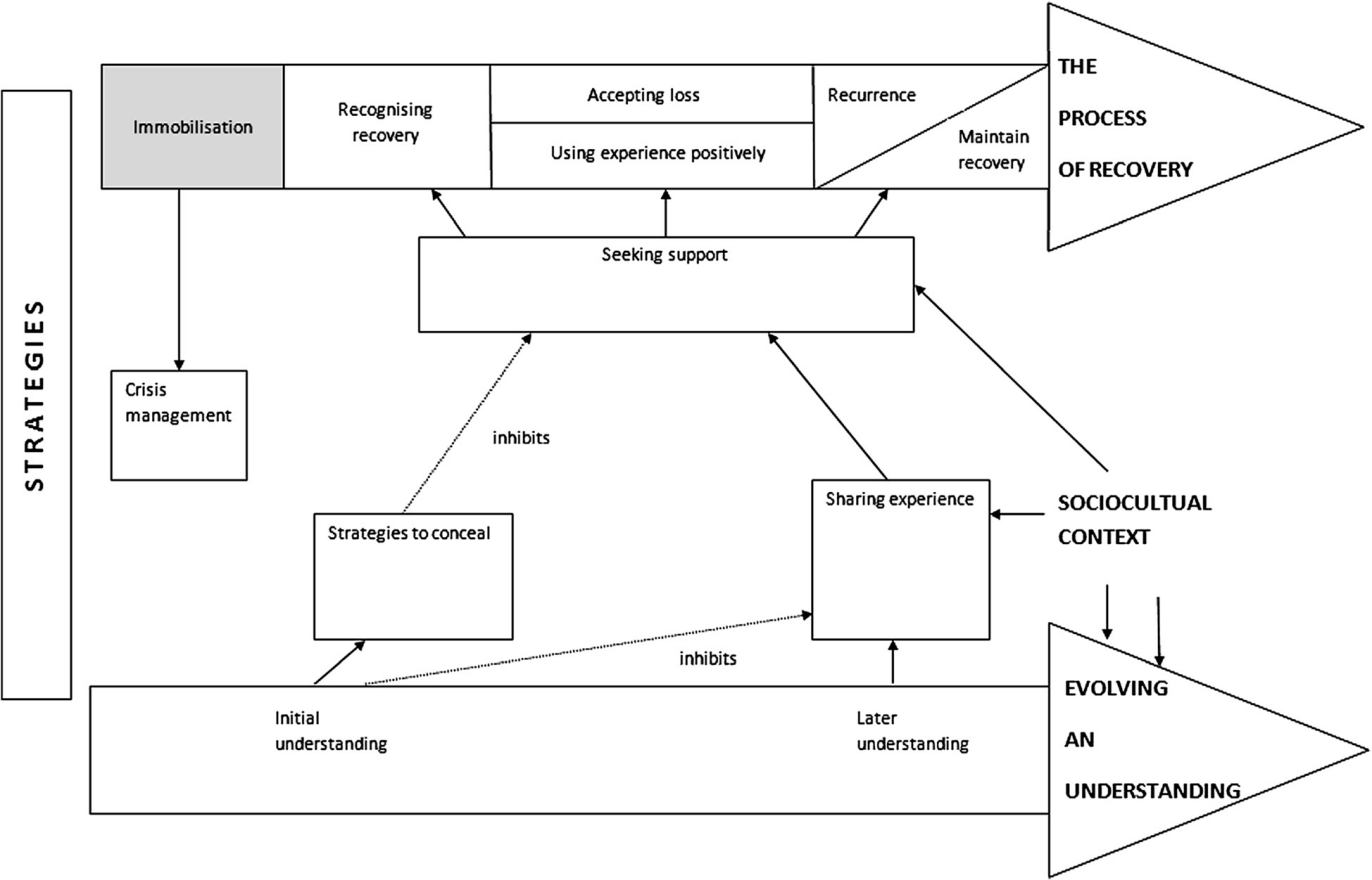
A diagrammatic representation of themes and their relationships.

A theory of four superordinate themes was developed from the data, including: (i) the process of recovery; (ii) evolving an understanding; (iii) strategies for recovery; and (iv) sociocultural context. The process of recovery and women’s understanding of their experience were conceptualised as parallel processes informing one another. A visual representation of categories and relationships is presented in Figure 1.

### The process of recovery

The first superordinate theme relates to the processes, which women identified as important for their recovery. Particular stages required negotiation before women could move on in the process of recovery.

### Immobilisation

Initially women discussed a period of immobilisation. Women did not view immobilisation as part of their recovery. However, it was important to include this stage because it had to be negotiated before recovery could begin and provided information about the point from which women were beginning their recovery. During immobilisation women were unable to make use of active strategies to recover, often relying solely on crisis management by health professionals.

Participant 9: “For the first few couple of weeks or few weeks I was there I was just too- I was just psychotic, I just needed to- that needed to get right really before anything could happen anyway.”

### Recognising recovery

Recognising recovery involved focusing attention on changes in experience which women considered to be indicators of recovery. This was facilitated by feedback from others because often other people noticed initial improvements. Once women had begun to recover, self-efficacy and hope increased and they felt able to implement more active strategies. Women discussed recognising their recovery in terms of “turning points:” significant positive changes in their situations which contributed to a more hopeful understanding of their experience, reinforcing their use of strategies and the process of recovery.

Participant 1: “I did actually I think for the first time I thought, “Maybe I can do this.”

Recognition of recovery and evaluating one’s position within the recovery process was something, which was ongoing, but not without obstacles. Women expressed uncertainty about their position with respect to recovery.

### Accepting loss

Although women found that many losses associated with psychosis were reversed during recovery, some remained, meaning recovery necessitated acceptance of these losses. This involved revising expectations in light of their beliefs about the consequences of their experience. Women expressed feelings of sadness, anger and guilt that they were unable to participate fully in caring for and bonding with their child. They expressed anxiety about the potential adverse consequences of the experience for their child’s development and described a painful reliving of their experience at times. Women who felt they could not risk subsequent pregnancies expressed anger, sadness and guilt that this choice had been taken away from them.

Participant 2: “It’s very painful now. You know, you see these babies and I can’t bear looking at babies.”

Women expressed their desire to reduce stigma associated with mental illness but were resigned to the fact that it would remain to some extent. They discussed how the experience had put a strain on their relationships. Although many women regained their sense of self during the process of recovery, others felt they had undergone a change that was likely to be permanent.

### Using experience positively

Although women acknowledged that their experience of psychosis had been extremely traumatic, it was important for them to find something positive in their experiences in order to move on in the process of recovery.

Participant 5: “I truly believe now, everything we go through can be used for good.”

Women discussed a renewed appreciation for positive things in their lives and an increase in qualities, such as empathy and patience. They discussed positive changes to relationships and felt passionate about wanting to help others experiencing similar difficulties, which was evidenced by women’s proactive attempts to effect changes. Whereas initially women had felt the experience was incongruent with their identity, using it positively facilitated acceptance of it into their narratives.

### Maintaining recovery versus recurrence

Women’s worry about recurrence and active attempts to maintain recovery suggest that they viewed the process of recovery as ongoing. Strategies used to maintain recovery were extensions of those used to recover from acute illness. Women discussed that the process of recovery was not smooth and that often periods of apparent recovery were followed by a worsening of their condition and, in many cases, a return to hospital. Women often linked these events with strategy use. Women often reflected that a recurrence might have been prevented, informing their evolving understanding of their experience and their use of strategies.

For many women, fear of recurrence strengthened their decision to have no further children. The minority who went on to have further pregnancies and children felt that their desire to have more children outweighed their fear of recurrence. They attempted to give themselves the best chance of staying well by seeking advice from professionals who had specialist knowledge and experience.

### Evolving an understanding

The second superordinate theme relates to women’s beliefs about their experience, which were found to influence their emotional responses, behaviours and their relationships with health-care providers and others. As women moved on in the process of recovery, their understanding of the experience was continually evolving with implications for the process of recovery and strategies used. Evidence that women’s understanding was dynamic included disparity in their descriptions over the course of their accounts.

### Initial understanding

Initially, women identified a mismatch between their expectations and experience of motherhood. Women discussed feeling devastated that they were unable to meet expectations of them as new mothers imposed by society, leading them to feel guilty and ashamed.

Participant 12: “I still had a real thing about…it felt like I was- it felt like I’d failed I suppose. I probably felt a bit guilty.”

Women experienced conflict between their sense of self and their perceived movement toward the role of “mentally ill person.” Women’s perceptions of this role were influenced by the socially constructed stereotype of someone with a mental illness imposed by society. If the perception was incongruent with their identities, it caused them shame and fear of stigma.

Participant 12: “Everybody’s worst nightmare in the world if they’re very honest with you, they would say, it would to lock- to be locked in a- in an asylum or a mental hospital because of the way it’s portrayed on telly and the white coats and padded cells and stuff like that.”

### Later understanding

Women evaluated their use of strategies and developed beliefs that their symptoms were predictable and controllable to some extent based on their behaviour. Awareness of their needs increased. Women developed an understanding that strategies, such as stress avoidance, contributed to progressing and maintaining recovery. Women weighed up these competing demands and some re-evaluated the importance of striving to meet role expectations. Mothers described experiences, which challenged their stereotype of “mentally ill person.”

Participant 12: “For a long time felt I had to blame something or somebody for it…um…and seeing that it can happen to somebody else in very much the same circumstances as me makes you think, “Well, it isn’t my fault. There was nothing I could have done about it.”

### Strategies for recovery

The third superordinate theme relates to strategies women used to recover based on their beliefs about their experience. Initially women felt powerless in relation to their symptoms, other people, the mental health system and society.

### Crisis management

When women felt unable to understand, control or predict their symptoms, they had no option but to rely on intervention from health professionals. This dependence created conflict for women who did not trust the professionals they were working with.

Participant 1: “The very people you reach out to help you then become almost like your enemy, you’re fighting against them and they’re the people that were supposed to help us.”

As women developed beliefs about the controllability of their symptoms and recognised recovery had led to increases in hope and self-efficacy, they were more motivated to experiment with strategy use.

### Strategies to conceal illness

Initial understanding motivated women to conceal or minimise their experience in relation to professionals and also their informal support networks. This included reporting thoughts and feelings that did not correspond to their subjective experience and resisting urges to engage in behaviours that they believed to be indicative of illness. Women were guided by beliefs about society’s expectations of a new mother.

Participant 3: “Trying to live up to these standards or what other people might expect from, you know, your idea of a good Mum.”

These strategies were also driven by a fear of hospitalisation and custody loss and were accompanied by a fear of exposure and feelings of guilt and shame. Women discussed examples in which the responses of others reinforced this strategy.

Participant 6: “When I was really bad soon after I had the diagnosis, it was just something that was…um…avoided, yeah. They wouldn’t ask me how I was. It’s like the whole stigma of mental illness mustn’t be talked about.”

### Sharing experience

As their beliefs about their experience evolved, women’s desire to avoid stress superseded their need to conform to personal, social and societal expectations. Women referred to feeling empowered as a result.

Participant 4: “I…possessions and things…I used to be really career orientated. I wanted to do well, I wanted to be [top of profession]. Now I don’t have…you know, maybe I will when he’s older. I don’t have any inclination to do that now.”

Women described experiences in which their beliefs about mental illness were challenged and this made them more able to accept the experience and use it positively. Over time they were more able to be open with others about their experiences and seek much needed support. Fear of stigma was replaced by a more resilient stance toward negative reactions of others and by a motivation to decrease stigma.

### Seeking support

Increased choice about disclosure allowed women to access support, including support from professionals, informal support networks and other women with similar experiences. Women emphasised how each stage of the recovery process could be facilitated by seeking support. For many women this was the most important factor in their recovery and there were many examples of women engaging in emotional expressions of gratitude to their family and friends.

Greater openness facilitated collaboration with a range of health professionals. Women discussed how the quality of relationships was an important determinant of support and discussed the properties of a relationship in which feelings of safety were enhanced, facilitating development of a positive relationship. These properties included health professionals giving women positive messages about ability to recover, empathic responses to their behaviour, and flexibility and responsiveness in the level of support.

Participant 4: “How they were flexible with me was really good. Um…you now, letting me do it in my own time.”

### Sociocultural context

The fourth superordinate category relates to the contextual factors in women’s lives. Women constructed and reconstructed their understanding of their experience based on their interactions with various sociocultural contexts, which were encountered during the recovery process. These contexts included the mental health system, informal support networks, women with similar experiences and researchers.

Although women’s beliefs incorporated aspects of the medical model, which dominates the mental health system, they were not restricted by it. Accounts were characterised by uncertainty regarding causes. Women believed that their experience was the result of multiple causes that combined to create a “perfect storm” or “domino effect,” leading to the selection of multiple strategies for recovery. Women stated that biological factors, such as hormones, chemicals and genetics, were important but believed that “it’s not the whole story.” They appeared to hold a stress-vulnerability model believing that they were biologically vulnerable to psychosis but that it was triggered by other factors.

Participant 2: “My brain doesn’t fit well with the birth process but then the sleep deprivation might have been the thing that, the switch that…the fuse that blew.”

Triggers discussed by women included sleep deprivation, stress, trauma, bereavement and lack of social support. Many women discussed difficulties associated with breastfeeding, including perceived pressure from professionals, contributing to anxiety and feelings of failure. Women varied in the emphasis that they placed on biological and psychosocial causes, although all women spent some time discussing both. Spiritual factors were discussed less often but had important implications for strategy use.

An important part of women’s interaction with the mental health system was receiving a diagnosis. Women discussed that receiving a diagnosis contributed positively to their understanding. It challenged beliefs that they were “going mad” and that there was no hope for recovery.

Participant 6: “Even though it was this thing you’d not heard of, it was a relief to know…it does exist, other people have had it before me and there are things that can be done.”

The circumstances surrounding diagnosis were important. Women discussed gaps in memory with respect to discussion around the meaning of the diagnosis. Therefore, they supplemented explanations given by health professionals by self-initiated information seeking, which was facilitated by diagnosis.

Not all women described receiving a diagnosis as a positive experience. One woman felt that the absence of a diagnosis was an advantage because professionals had to be responsive to her individual needs rather than making assumptions based upon a label. It is interesting to note that this particular woman did not use information seeking as a strategy, because she felt the costs outweighed the benefits. Therefore, the role of diagnosis in facilitating the use of this strategy was not relevant.

As well as the impact on women’s beliefs about their experience, sociocultural context also had important implications for strategy use. Women discussed how their evolving understanding allowed them to overcome fear of stigma and feelings of shame and hopelessness to enable more openness and support seeking. Women discussed non-conformity with expectations by postponing or limiting their employment or withdrawing from stressful relationships. However, these changes were made possible by financial security and strong informal social support networks. It is hypothesised that women from lower socioeconomic status (SES) groups with impoverished social support may be less able to make such changes.

Participant 10: “I felt quite isolated at times… I was so desperate to come back up here… So we moved house, we got married and I tried to go back [laughs] to work… I knew as soon as I went back to work that I wasn’t well enough to be there.”

## Discussion

This is the first study to investigate recovery in women with experience of psychosis following childbirth in order to develop a theoretical understanding of this process using grounded theory. The themes generated confirm and extend findings from previous studies investigating recovery. We found that our sample embarked on a complicated process of recovery, in which these women were active agents rather than passive recipients of “treatment.” The strategies they used to recover were inextricably linked with their evolving understanding of illness and recovery. Women considered both biological and psychosocial factors to be important for understanding their experience and noted the importance of other people’s support in their recovery. In addition, their beliefs about their experience influenced their emotional and behavioural responses, as well as their relationships with health professionals [16], consistent with theoretical models developed in physical health, such as the self-regulation model (SRM) [17].

The women in our study held uncertain beliefs that evolved over time. They discussed changes in their understanding of their experience even years after the event. Therefore evolving an understanding was viewed as a process that ran in parallel with the process of recovery. Initially, the perceived uncontrollability and unpredictability of symptoms challenged women, negatively impacting self-efficacy and hopelessness. According to Kinderman et al. [18], people with mental health problems have beliefs that are confused, inconsistent or contradictory and that changes in the course of psychological difficulties may influence people’s beliefs about their experiences. Our findings also highlight the importance of women’s interactions with others in their evolving understanding of their experience.

Current approaches to illness beliefs in physical health assume that people distinguish between the illness and themselves. Women struggled with this distinction at times. For example, as new mothers, they wondered whether aspects of their experience were attributable to psychosis or the new experience of motherhood. Women also talked about permanent changes in their sense of self that had resulted from the experience, both positive and negative. An important part of women’s recovery was using their experience positively, which facilitated incorporation of their experience into their narrative and sense of self. This corroborates the finding of Kinderman et al. [18] that participants did not separate their experience from their identity and that some participants linked their experience to a sense of purpose.

Bondi and Burman [19] argued that labelling women with mental illness contributes to their oppression and subordination; however, a diagnosis can have both a positive and negative impact on an individual [20]. Whilst the restrictive aspects of a diagnosis were recognised, the majority of women found that it enabled them to develop less distressing interpretations of their experience, echoing the finding that women with postnatal depression (PND) felt grateful when they discovered they had a diagnosable illness as opposed to being “crazy” or “bad mothers” [21]. Women emphasised the importance of the context in which a diagnosis is given. If a diagnosis is accompanied by a lack of information, this can cause disempowerment and hopelessness [22]. Indeed women in the current study reported initially feeling powerless in relation to their symptoms, other people, the mental health system and society.

Women believed that support from professionals was an essential component of recovery, substantiating reports from the PND recovery literature [23]. However, they spent a large proportion of their time discussing barriers to accessing this support, finding it difficult to trust professionals who they perceived to have the power to hospitalise them and question their ability to parent their child. This power imbalance has been recognised elsewhere [24], motivating women to conceal aspects of their experience, particularly in relation to possible hospitalisation and custody loss [25]. The importance of staff having an optimistic and hope-inducing view about the ability of people to find meaning in their experiences has been recognised [4].

Although women’s beliefs about their experiences were clearly influenced by their interactions with services, which are dominated by a knowledge base that ‘medicalizes’ distress [26], this did not restrict their accounts. Women spent a lot of time discussing their beliefs about what caused psychosis. Their accounts were characterised by uncertainty and they discussed a combination of causes, both biological and psychosocial. This contradicts the finding by Robertson and Lyons [27] that “women spent little time discussing causes, as they all consistently and concisely described the cause of their illness as biological” (p. 418). It is possible that women’s accounts in Robertson and Lyons’ study were influenced by their prior involvement in a genetics study. This discrepancy is important because it has been reported that women have been critical of the way in which mental health professionals dismiss the role of contextual factors that may influence mental health [28], presenting a barrier to engagement with services. Professionals have been advised to take precautions against limiting change by imposing an explanatory illness model when other models may be more helpful for the individual [4]. Women’s uncertainty about what caused their illness supports the finding that people have difficulty in conceptualising their mental illness [18].

Women in the current study, whilst initially driven to conform to society’s expectations of them by concealing their illness experience, developed an understanding that allowed them to re-evaluate their need to conform. For example, women with PND choose to remain silent due to concerns about exposure to the public compounded by high expectations of themselves [29]. Our sample discussed initially feeling reluctant to share their experiences with others which presented a barrier to accessing such support. They illuminated this process further, because they also discussed becoming more able to share their experiences with others as their desire to help other women and fight stigma became more important than their need to prevent exposure. However, the freedom to do this was mediated by social support. Women discussed how their informal social support networks reduced the perceived threat of hospitalisation and custody loss imposed by services. Women stressed support from friends and family as the most important factor in their recovery. The importance of the changing nature of relationships with both professionals and informal support networks for recovery has also been documented [23,30]. It has been argued that women with fewer economic resources may have less social support [31]. Culture also plays a role in the nature of support, which families provide to relatives with psychosis, which in turn appears to be related to the course of illness [32]. However, choice can be constrained by organisational, clinical, economic or attitudinal factors [33].

The current findings corroborate reports that people experience recovery as a gradual and uneven process involving turning points and milestones [2]. Recovery has been characterised as an ongoing process rather than an end result since the earliest accounts of recovery from mental distress in the 1980s [34,35]. Discrepancy in women’s accounts of their stage of recovery and anxiety regarding recurrence may be partly explained by the tension between different recovery styles, such as ‘sealing over,’ in which the significance of symptoms is minimised as a way of coping, and ‘integration’ [36].

The process of recovery began when women moved from an initial point of immobilisation to recognising the first signs of recovery. The concept of immobilisation is similar to the idea that the initial stage of recovery involves a period of withdrawal that is beneficial. Our findings highlight the key role of other people in enabling women to recognise their achievements, heightening motivation.

Whilst the use of strategies did give women a sense of control over their recovery, some aspects of their experience could not be changed and required acceptance. Loss has been recognised as part of the normal adjustment process for new mothers [37] and in relation to PND [38]. Pertinent to women in the current study were feelings of anger and sadness caused by the perception that choice around subsequent pregnancies had been taken from them, echoing the findings e.g., [27].

### Methodological limitations

Although the aim was to recruit women representative of different backgrounds, the entire sample in the present study was white British, therefore findings may not be transferable to mothers from other cultures. Although cross-cultural research into postnatal illness is imperative, because customs, rituals and social responses to motherhood vary between cultures [39], women who volunteer to take part in qualitative research tend to be white and from higher socio-economic status groups [40]. Nonetheless, cultural differences have been reported for women with PP [41], including differences in symptoms [42] and differences in beliefs about causes of the experience and attitudes to treatment [43].

Recruiting women from sources other than mental health services allowed exploration of biopsychosocial models of illness compared with a previous study [27], because involvement with services and interventions inevitably shape the beliefs women hold about their experiences. For some women, many years had passed since their experience of psychosis so their retrospective accounts may have been significantly affected. These women were included because it was necessary to include women at all stages of the recovery process. Although all women reported formal diagnoses of psychoses following childbirth, this was not verified for women recruited by advertisement. However, the demographic data collection tool included a symptom checklist and all women reported symptoms consistent with a diagnosis of psychosis following childbirth.

### Clinical implications

The current findings highlight a number of important implications. Women’s recovery depends upon the development of a useful understanding of their experiences. Professionals can facilitate this by presenting information at different stages of recovery to optimise assimilation. Informal support networks could be supported to assist with this process. Health professionals should focus on the following: (i) signs indicative of psychosis following childbirth to enable prompt recognition, (ii) women’s subjective experience of psychosis and formulation of challenging behaviour to facilitate empathic responding, and (iii) impact of one’s own values on women’s ability to recover. The quality of the relationship between women and health professionals needs a central focus. Giving women positive messages about their ability to recover, conveying a compassionate understanding of women’s often challenging behaviour and responsiveness to women’s changing level of need facilitate development of a positive working relationship. Given the importance of social support from informal support networks and the strain placed on these relationships as a result of psychosis, interventions aimed at supporting family members are likely to be relevant and useful. Given the value of meeting other women with similar experiences, professionals should assess women’s readiness to use this support and signpost appropriately.

## Conclusions

This study highlights a complex process of recovery from psychosis following childbirth, which is ongoing, even many years after the experience. The role of other people, including professionals, in this process, is central. Sensitivity to a woman’s position in the process of recovery has the potential to facilitate professionals in assessing readiness for different types of intervention.

## Competing interests

The authors declare that they have no competing interests.

## Authors’ contributions

LM carried out the study, led on the analysis and drafted the initial manuscript. AW oversaw the whole research process and contributed significantly to the study design and the final manuscript and its revision. SP offered her expertise in the analysis and significantly contributed to the final manuscript. AWie assisted with recruitment of participants and helped to draft the manuscript. All authors read and approved the final manuscript.

## Pre-publication history

The pre-publication history for this paper can be accessed here:

http://www.biomedcentral.com/1471-244X/13/341/prepub
